# A Brief Notice of a Highly Malignant Disease

**Published:** 1847-07

**Authors:** B. H. Pearson

**Affiliations:** Powelton, Ga.


*A brief notice of a highly Malignant Disease.* By B. H. Pearson, M. D., of Powelton, Ga.—Dec. 5, 1846, was called to see Green, a boy about 13 years of age, belonging to the Rev. Mr. ———. On or about the 1st of October he had a slight attack of fever, from which he gradually recovered under domestic prescriptions, so as to be able to resume his work on the plantation. He seemed, however, not to recover strength, but appeared weaker every day, until he finally left off work again, although still not confined to the house. He complained of great prostration and want of appetite. This, his master attributed to his having over-strained himself in carrying home his cotton, being extremely active, and picking more cotton than he could well carry; and for which he prescribed an occasional dose of Cook's pills, and tartar emetic ointment to the spinal column. I found him greatly emaciated; pulse extremely feeble, 175, rather irregular; the sounds of the heart were very weak, yet natural; respiration was feeble; no abnormal sounds; there was slight cough, with frothy expectoration in small quantities; appetite much impaired; bowels a little loose; stools dark; slight tenderness of the lower cervical vertebræ. Prescribe blue mass gr. j. at night, cups over the cervical vertebræ, and an easily assimilating diet.

9th.—Green says he is better, which continued to be his answer, when asked how he was, until the day of his death. A careful examination showed ***slight*** dulness under the left clavicle; bowels natural; no other change in the symptoms. Upon enquiry, I find he descended from syphilitic parentage, his grandfather, on his mother's side, having had that disease; and also that most of the male children from the same descent died in infancy. Prescribe hydriodate of potash grs. ijss. three times a-day, which was gradually increased to grs. iv. This was persevered in for about three weeks, when, seeing no improvement, but rather a gradual increase of his cough and his weakness, a resort was had to tonics, quinine, and stimulating expectorants, but with no benefit except relieving his cough a little at night.

Feb. 14th.—A neighbouring physician was called in, who pronounced his disease to depend on torpidity of the nutritive system. Prescribed hydriodate of potash grs. iij. three times a-day, under which prescription he remained until his death, which took place the last of February, being confined to his bed but three or four days before he died. He complained of no pain except a neuralgic affection of his knees for about a fortnight, and soreness of his hips from lying in bed. His bowels remained natural to the last.

*Post mortem examination fifteen hours after death.* Body extremely emaciated. Upon laying open the cavity of the thorax, the left pleura was found adherent throughout its whole extent; the heart and lung upon this side was perfectly studded with tubercles, of a cheese-like consistency, about the size of small buck-shot; the right lung was tuberculous, but not to the same extent as the left; a few tubercles on the upper surface of the liver, otherwise it was tolerably healthy; the spleen and peritoneum were equally affected; the pancreas, stomach and bowels healthy.

This case is given as an example of several occurring in the same family, four of whom have died, one is now at the point of death, and the disease seems to be extending to other families. I have not had an opportunity of seeing those sick in other families yet, but doubt not from the description of the symptoms that they are affected in the same way.

Thus far every one who has been attacked has died in a time varying from five weeks to four months—their symptoms varying in some particulars. One's bowels were badly affected for several weeks before death. In another, a large vomica bursted, and considerable matter was coughed up on the day preceding her death, which was probably the immediate cause of it. They all seemed to be taken sick by surprise, and died thinking they were getting well, except the mother of the family, who lived but five weeks after she first began to complain, and but two after she first felt sick enough to take to her bed, and she supposed she was "tricked."

Dr. Terrel, of Sparta, recommends the use of iodine to the remaining members of the family, as the only means of preventing the extension of the disease. Possibly it might be of service if used in season. But the probability is, that the disease may be advanced to an incurable state before the first symptoms appear,—and besides it is so insidious in its approach, that it is some time before the patient knows the nature of the attack. A disease similar to this prevailed some years ago in Maj. ———'s family, of Wilkes county, and between twenty and thirty died. I hear also it is prevailing in Tennessee, to an alarming extent in some neighbourhoods.—*Southern Med. and Surg. Journ.*

